# Kidney-Related Outcome in Cardiorenal Syndrome Type 3

**DOI:** 10.1155/2022/4895434

**Published:** 2022-02-07

**Authors:** Kim Drubel, Benedikt Marahrens, Oliver Ritter, Daniel Patschan

**Affiliations:** Klinik für Kardiologie und Nephrologie, University Hospital Brandenburg, Medical School of Brandenburg, Brandenburg, Hochstraße 29, 14770 Brandenburg, Germany

## Abstract

**Methods:**

A single-center, retrospective and observational trial. All subjects with positive AKI alert, treated at the University Hospital Brandenburg between January and December 2019, were evaluated. Definition of CRS type 3 was according to predefined criteria. The three endpoint categories were in-hospital death, dialysis, and recovery of kidney function.

**Results:**

. A total number of 1,334 AKI alerts were screened. Finally, 95 subjects received the diagnosis CRS type 3. The survival rates were 47.1% (females) and 43.6% (males). 46.8% of affected females and 33.3% of the males required dialysis therapy. Complete recovery at the time of discharge occurred in 35.8%, and no recovery at all was found in 54.7%.

**Conclusions:**

. All three predefined study endpoints, the mortality, the prevalence of dialysis, and the percentage of subjects without recovery of kidney function, were notably high. Therefore, AKI patients with imminent or established cardiac complications require the highest attention of nephrologists in charge.

## 1. Introduction

In 2008, Ronco et al. introduced the concept of cardiorenal syndromes (CRS) [1]. The authors differentiated between five distinct CRS types, all characterized by functional/structural affection of both the heart and kidney in an acute or chronic manner. The general concept was (and still is) to emphasize the inter-/multidisciplinary character of the diseases, not only from a pathophysiological but also from a therapeutic perspective.

In CRS type 3, acute kidney injury (AKI) [2] induces and, sometimes, even perpetuates acute cardiac pathologies such as arrhythmias with or without cardiac decompensation or the latter due to other causes (e.g., aggravated hypertension and fluid retention) [3]. Epidemiological data on CRS type 3 are limited. According to the definition of the syndrome, any type of cardiac complication due to AKI allows the diagnosis. De Abreu et al. [4] performed a retrospective investigation in 129 subjects treated at the ICU between January 2006 and January 2008. Fifty-two individuals developed AKI (40%), and cardiac arrest was identified as the cause of death in 20.3%. The actual incidence of AKI-associated cardiac complications (and, thus, of CRS type 3), however, ranges much higher for sure since other consequences than cardiac death must be considered also: pulmonary congestion, arrhythmias of various etiology, pericarditis, and coronary artery insufficiency.

AKI remains a significant problem in hospitals worldwide, and the incidence has been documented to vary between 20 and 30% [5]. Under intensive care conditions, however, the incidence may even exceed 50% [6]. In 2015, Hoste et al. published a multinational, prospective trial on AKI epidemiology and outcomes in ICU-treated patients (AKI-EPI study [7]). In total, 139 intensive care units participated, providing more than 2,000 eligible patients from which, finally, 1,802 were included in the analyses. It turned out that AKI dramatically increased the risk for death from 4.7% (no AKI) to 24% (AKI) (*p* < 0.001). The mortality risk gradually increased with increasing AKI severity according to KDIGO [2] (odds ratio stage 1 : 2.19; stage 2 : 3.88; and stage 3 : 7.18). Although some progress has been achieved in terms of improving the prognosis of AKI subjects, at least in middle-to-higher-income countries [8], the overall mortality risk in the short-term has only marginally been improved over the last 20 years. Also, surviving subjects are at higher risk for chronic kidney disease (CKD) later in life [9–11]. In 2013, Lewington summarized AKI-related outcomes and costs from a global perspective [12]. It was emphasized that the death rate of all AKI subjects exceeds the death rate of heart failure, diabetes, and breast and prostate cancer combined. It was, therefore, more than justified that the authors introduced AKI as a ‘silent killer.'

The principal aim of cardionephrologists is to improve the prognosis of cardiorenal patients. In order to achieve this goal, essential questions need to be answered: are patients with acute onset CRS, namely, types 1 and 3, at higher risk for death/dialysis than AKI subjects in general? Is the chance of renal recovery lower than in AKI in general? The current article intended to find answers to these questions. Its exclusive focus is on CRS type 3.

## 2. Methods

### 2.1. Design

The investigation was conducted as a single-center, retrospective and observational study. It was performed at the University Hospital of Brandenburg of the Brandenburg Medical School. The local ethics committee of the Brandenburg Medical School formally approved the study (No. E-01-20200602). The committee decided that it was not required to obtain written consent due to the retrospective design. All patient-related information was extracted from the local hospital information system (MEDICO^®^, CompuGroup Medical). The observational period lasted from January 2019 until December 2019. Based on an electronic algorithm, every patient with an increase of serum creatinine according to criteria 1 or 2 of the ‘KDIGO clinical practice guidelines for acute kidney injury' from 2012 [2] was screened. Patients were included if they met the definition criteria for CRS type 3 (see below). Additional inclusion criteria were age>=18 years and in-hospital treatment for a minimum of 2 days. If AKI occurred more often than once per single in-hospital treatment period, only one AKI episode was considered. Not included were patients with established CKD 5D or with CHF stage 4 according to the NYHA classification.

### 2.2. Definition of CRS Type 3

The diagnosis of CRS type 3 was made if AKI according to the KDIGO guideline [2] occurred prior to a cardiac event with acute onset. A cardiac event with acute onset was diagnosed if one or more out of three symptoms/findings were present: (I) dyspnea including symptoms of congestion, (II) radiographic findings of pulmonary fluid accumulation, or (III) certain echocardiographic findings: reduced left ventricular ejection fraction, local wall motion abnormalities, and valve dysfunction of the left ventricle (at least grade 2). The definition criteria were quite similar as used in a recently published study by our group: ‘Risk factors and outcome variables of cardiorenal syndrome type 1 from the nephrologist's perspective' [13]. The mentioned study focused on CRS type 1 instead, and the differentiation between CRS types 1 and 3 was made after considering the dynamics of AKI onset. If AKI comprehensibly occurred secondary to cardiac complications, the definition of CRS type 1 was fulfilled. Otherwise, CRS type 3 was diagnosed.

### 2.3. Endpoints

The primary endpoint was in-hospital survival. Secondary endpoints were the need for dialysis and the recovery of kidney function until discharge from the hospital. Complete renal recovery was diagnosed if the last eGFR was 10% higher than the lowest eGFR. If the last eGFR was higher than the lowest value during the treatment course but not by more than 10%, we diagnosed incomplete recovery.

### 2.4. Statistics

Comparisons between two groups were performed with the chi square test for categorial data. Noncategorial data were initially tested for normal distribution by using the Kolmogorov–Smirnov test. Data with normal distribution were compared with Student's test, and data lacking normal distribution were compared with the Mann–Whitney test. A *p* value of below 0.05 was stated as statistically significant. Results are either given in percent or as mean+/−SD or SEM as indicated.

## 3. Results

During the observational period, a total number of 1,334 AKI alerts were screened. Of these 1,334 AKI alarms, 779 were dual alarms or multiple alarms. Each patient was included in the evaluation only once. Effectively, 555 of 1,334 AKI alarms were evaluated more in detail. Out of these 555 patients, 95 patients fulfilled the diagnosis criteria of CRS type 3 according to the methods section.

### 3.1. Baseline Characteristics

A total number of 95 individuals (female: 42; male: 53) were diagnosed with CRS type 3 according the criteria defined in the methods section. The mean age of all individuals was 79.6+/−10.5 years. The mean duration of in-hospital treatment was 21.1+/−17.3 days. The AKI stages according to KDIGO [1] were 1–20%, 2–40%, and 3–40%. All baseline data are summarized in Table 1.

### 3.2. Outcome Analyses

Three categories were defined as outcome parameters: survival, need for renal replacement therapy (RRT-dialysis), and recovery of kidney function or renal recovery. Every category was tested for the following variables: gender, age, duration of in-hospital stay, AKI stage according to KDIGO [2], initial, peak, and last serum creatinine, initial, peak, and last serum sodium and potassium concentrations, respectively, initial and peak NT-proBNP and CRP, vasopressor therapy, invasive ventilation, arterial hypertension, diabetes mellitus, obesity, preexisting coronary artery disease (CAD) and chronic heart failure (CHF), chronic obstructive pulmonary disease (COPD), hyperuricemia, and history of neoplasia.

### 3.3. Survival

**Figure 1:**
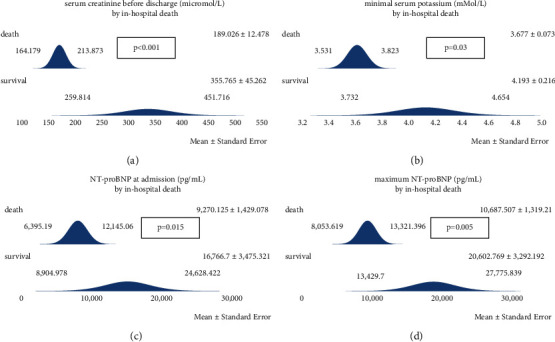
Regarding the outcome parameter ‘survival,' four variables were significantly different between surviving and nonsurviving subjects. (a) The last serum creatinine concentration measured, either before death or discharge from the hospital, was higher in survivors. (b) The minimal serum potassium level during the stay at the hospital was higher in survivors. (c), (d) Both the initial and the maximum NT-proBNP were also higher in surviving patients (data are represented as mean+/−SD).

The survival rates were 47.1% in females and 43.6% in males. The following variables significantly differed between surviving and nonsurviving individuals: serum creatinine before discharge (higher in survivors), minimal serum potassium (higher in survivors), and initial and peak NT-proBNP (both higher in survivors). The detailed numerical results and all respective *p* values are shown in Table 2.  Figure 1 illustrates all differences that reached the level of statistical significance.

### 3.4. Dialysis

**Figure 2:**
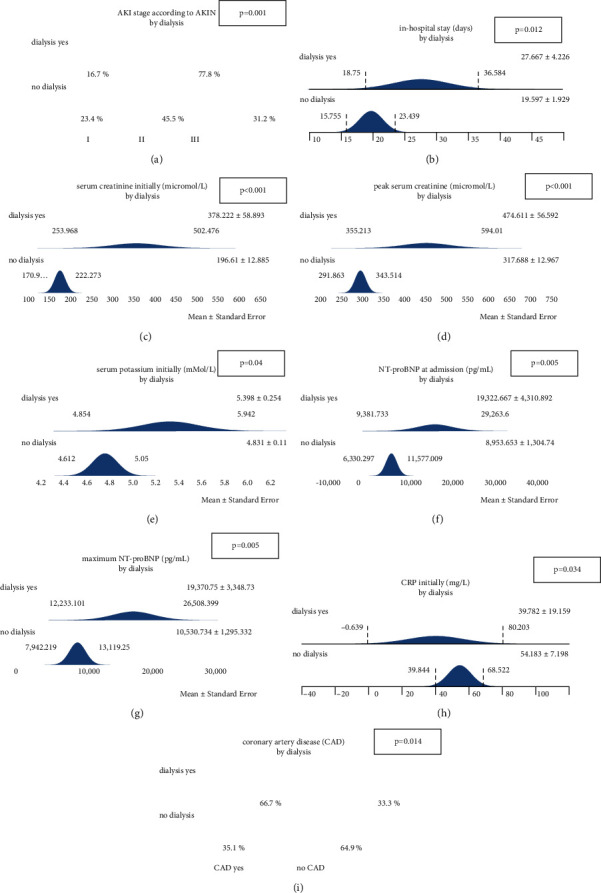
Several variables significantly differed between subjects with versus without the need for dialysis therapy (‘dialysis yes' versus ‘no dialysis'). (a) AKI stage 3 more prevalent in ‘dialysis yes.' (b) In-hospital stay (longer in ‘dialysis yes'). (c), (d) Initial and peak creatinine higher in ‘dialysis yes.' (e) Initial serum potassium higher in ‘dialysis yes.' (f), (g) Initial and maximum NT-proBNP higher in ‘dialysis yes.' (h) Initial CRP higher in ‘no dialysis.' (i) Coronary artery disease more prevalent in ‘dialysis yes' (data are represented as mean+/−SD).

The term ‘dialysis' is employed instead of renal replacement therapy (RRT). In total, 46.8% of affected females and 33.3% of the males required dialysis therapy. Several variables significantly differed between subjects with versus without the need for dialysis therapy (‘dialysis yes' versus ‘no dialysis'): AKI stage 3 more prevalent in ‘dialysis yes,' duration of in-hospital stay (longer in ‘dialysis yes'), initial and peak creatinine (both higher in ‘dialysis yes'), initial serum potassium (higher in ‘dialysis yes'), initial and maximum NT-proBNP (higher in ‘dialysis yes'), initial CRP (higher in ‘no dialysis'), and coronary artery disease (more prevalent in ‘dialysis yes').  Table 3 and Figure 2 summarize all results in detail.

### 3.5. Renal Recovery

**Figure 3:**
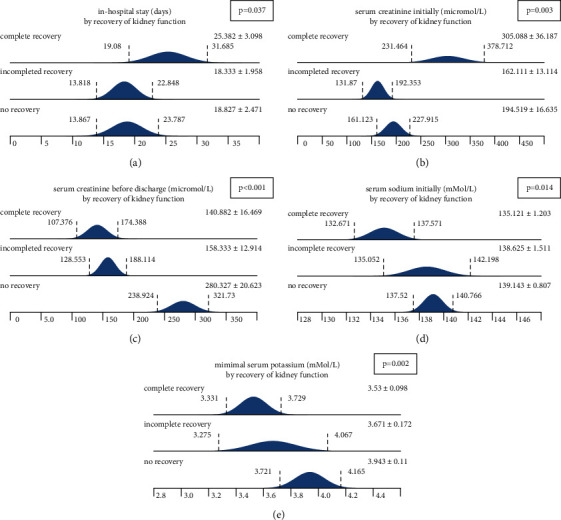
Five variables differed between the subcategories ‘no recovery,' ‘incomplete recovery,' and ‘complete recovery.' (a) In-hospital treatment time (longest period in subjects with complete recovery). (b) Initial serum creatinine sodium (highest concentration in subjects with complete recovery). (c) Serum creatinine before discharge (highest concentration in subjects without recovery). (d) Serum sodium initially (lowest concentration in the ‘complete recovery' subgroup). (e) Minimal serum potassium (lowest concentration in subjects with complete recovery) (data are represented as mean+/−SD).

Regarding renal recovery, three recovery stages were defined: no recovery, incomplete recovery, and complete recovery. Complete recovery at the time of discharge occurred in 35.8%, and no recovery at all was found in 54.7%. The respective definitions are given in the methods section. Five variables were unequally distributed between the stages: in-hospital treatment time (longest period in subjects with complete recovery), initial serum creatinine sodium (highest concentration in subjects with complete recovery), serum creatinine before discharge (highest concentration in subjects without recovery), serum sodium initially (lowest concentration in the ‘complete recovery' subgroup), and minimal serum potassium (lowest concentration in subjects with complete recovery).  Table 4 and Figure 3 summarize all results in detail.

## 4. Discussion

From the nephrologist's perspective, three parameters are particularly interesting in CRS type 3: in-hospital mortality, prevalence of renal replacement therapy (dialysis), and renal recovery until discharge. Further parameters of interest were, of course, the mortality and renal recovery during follow-up. However, these data are not available yet.

### 4.1. Mortality

The in-hospital mortality was 47.1% in females and 43.6% in males. Numerous studies analyzed outcome variables in AKI in general. Selby et al. [14] reported a mortality of 23.8%, and Uchino et al. [15] identified death to occur in 14.8% of patients with transient azotaemia and in 23.8% of subjects with acute tubular necrosis. In 2006, Waikar et al. [16] published a large-scale longitudinal study, extracting data from the ‘Nationwide Inpatient Sample (NIS)' database. More than one milion individuals with documented AKI (herein, ARF, acute renal failure) were identified between 1988 and 2002. The mortality significantly decreased over time (40.4 to 20.3%; *p* < 0.001). Comparable dynamics were reported in 2016 [17]. In a retrospective observational study, data were collected from the ‘Hospital Episode Statistics' (HES) repository, which covers the entire English National Health Service. In total, 1,136,167 AKI events were identified between 1998 and 2013, and the so-called case-fatality (mortality) decreased from 42.3% to 27.1% (*p* < 0.001). In a 2019 published retrospective study, the AKI-associated mortality increased from AKI stage 1 to 3 according to KDIGO (5.1% > 13.7% > 24.8%) [18]. Finally, recently published data from our hospital showed an in-hospital mortality of 26% [19]. In summary, the overall AKI-associated mortality does not exceed 30% nowadays. In the current study, four variables significantly differed between survivors and nonsurvivors: serum creatinine before discharge, minimal serum potassium during hospital treatment, and initial and peak NT-proBNP. Surprisingly, all four parameters were higher in survivors. These findings are hardly explainable. Survivors and nonsurvivors did not differ in gender, age, duration of in-hospital treatment, or AKI stage according to KDIGO [2]. Regarding NT-proBNP, it may be argued that relative or absolute hyperhydration due to cardiac insufficiency potentially acts renoprotective. On the other hand, serum creatinine before discharge was significantly higher in survivors also. In addition, surviving individuals required RRT not less often than nonsurvivors (17.6 vs. 19.2%; *p*=0.88). Since many years, NT-proBNP has been known to be survival predictive in chronic heart failure [20]. Momentarily, any reasonable explanation for higher NT-proBNP levels in survivors is nothing but speculative in nature. The higher serum creatinine before discharge in surviving individuals requires an even stricter follow-up management after discharge from the hospital. AKI of various etiologies increases the CKD risk [11], and surviving CRS type 3 subjects should receive regular follow-ups by a specialized nephrologist without doubt.

### 4.2. Dialysis

In total, 46.8% of affected females and 33.3% of the males required dialysis therapy, and the difference was not statistically significant. Selby et al. [14] reported a dialysis prevalence of 3.4% in 2,619 AKI patients observed during a period of 9 months. The prospective, multinational AKI-EPI study, performed in 97 intensive care units, revealed that renal replacement therapy became mandatory in 13.5% of all included subjects and in 23.5% of ICU-treated patients with AKI [7]. Several variables were significantly higher in dialysis-requiring as opposed to dialysis-native subjects: initial and peak serum creatinine, initial serum potassium, and initial and peak NT-proBNP. In contrast, the initial CRP was lower. AKI stage 3 was diagnosed more often in RRT-requiring patients; in addition, the in-hospital treatment time was longer in dialyzed subjects. Finally, the prevalence of CAD was higher in subjects that required RRT. The high dialysis prevalence in CRS type 3 is as surprising as the dramatically increased mortality. In 2011, Fabbian et al. [21] reported epidemiological characteristics and outcome variables of CRS patients in a retrospective manner. More than 500 subjects were included, and CRS type 3 was diagnosed in ∼20%. Only a small proportion (1%) of all subjects received dialysis therapy at all. An exact determination of the actual percentage of patients with the need for dialysis is most likely more feasible under prospective study conditions.

### 4.3. Renal Recovery

As summarized by Hoste et al. [8], post-AKI recovery of kidney function is an outcome parameter of fundamental importance. Kellum et al. [22] showed a dramatically reduced survival probability in post-AKI subjects (septic AKI) without recovery at 1 year after the incident. The same leading author published a retrospective analysis performed in 16,968 critically ill subjects with AKI stages 2 and 3 according to KDIGO [2]. No reversal of kidney function at all was documented in 26.5% [23]. We found no recovery at all in almost 55% of the patients (54.7%), which was even higher than the highest percentage reported in septic AKI subjects receiving usual care (35.4% [22]). Among the variables that differed between no recovery, incomplete recovery, and complete recovery, one variable was particularly interesting: initial serum sodium (highest concentration in the no recovery group). Hyponatremia, for instance, has been shown to be associated with poor short- and long-term outcomes in heart failure patients [24, 25]. Also, hyponatremia was documented as predictive in CRS type 1 [26]. On the other hand, Peres et al. [27] identified hypernatremia as an independent mortality risk factor at the ICU, and another study showed hypernatremia as predictive for sepsis-induced AKI [28]. A PUBMED-based search for the terms ‘hypernatremia' and ‘cardiorenal syndrome,' however, reveals no results at all (08/2021). Thus, hypernatremia may potentially indicate CRS type 3 patients with a lower chance for renal recovery. It, nevertheless, needs to be mentioned that serum sodium was not elevated over the normal upper range in any recovery subcategory, but the highest concentrations were found in subjects without recovery at all. Whether established hypernatremia is potentially recovery predictive or not needs to be evaluated in a higher number of CRS type 3 subjects.

The most important limitation of our study is for sure the retrospective and, thus, observational design. To retrospectively identify CRS type 3 in a reliable manner is difficult, if not impossible in all incident patients. Another limitation is the exclusion of medication-related informations. Drugs that are used for heart failure treatment (e.g., ACE inhibitors) potentially affect the renal prognosis in AKI. The same applies for other medications such as nonsteroidal anti-inflammatory drugs or certain types of antibiotics.

Nevertheless, the prognosis of CRS type 3 patients is at least serious, if not poor. All three predefined study endpoints, the mortality, the prevalence of dialysis, and the percentage of subjects without recovery of kidney function, were inacceptably high. Therefore, AKI patients with imminent or established cardiac complications require the highest attention of nephrologists in charge. In addition, further research must address the question how AKI patients at risk for CRS type 3 may be identified as soon as possible.

## Figures and Tables

**Table 1:** Baseline characteristics of all patients included.

Variable	Baseline characteristics
Gender (females/males)	42 (44.2%)/53 (55.8%)
Age (mean years+/−SD)	79.6+/−10.5
In-hospital stay (mean days+/−SD)	21.1+/−17.3
AKIN stage (1/2/3)	19 (20%)/38 (40%)/38 (40%)
Initial serum creatinine (micro-Mol/L+/−SD)	231+/−163
Peak serum creatinine (micro-Mol/L+/−SD)	347.4+/−157.2
Serum creatinine before discharge (micro-Mol/L+/−SD)	218.8+/−141.4
Initial serum sodium (mMol/L+/−SD)	137.6+/−6.2
Minimal serum sodium (mMol/L+/−SD)	135.1+/−6
Peak serum sodium (mMol/L+/−SD	144.1+/−5.5
Initial serum potassium (mMol/L+/−SD)	4.9+/−0.9
Minimal serum potassium (mMol/L+/−SD)	3.7+/−0.7
Peak serum potassium (mMol/L+/−SD)	5.2+/−0.8
Initial NT-proBNP (pg/mL)	10,562+/−10,395
Peak NT-proBNP (pg/mL)	12,298+/−11,505
Initial CRP (mg/L+/−SD)	51.4+/−66.4
Peak CRP (mg/L+/−SD)	134.1+/−105.5
Vasopressors (*n* and %)	8 (8.4%)
Invasive ventilation (*n* and %)	12 (12.6%)
Arterial hypertension (*n* and %)	87 (91.6%)
Diabetes (*n* and %)	54 (56.8%)
Obesity (*n* and %)	52 (55.3%)
Preexisting CHF (*n* and %)	26 (27.4%)
Preexisting CAD (*n* and %)	39 (41.1%)
COPD (*n* and %)	18 (18.9%)
Hyperuricemia (*n* and %)	23 (24.2%)
History of cancer (*n* and %)	26 (27.4%)
Nephrotoxic drugs (*n* and %)	
NSAIDs	33 (34.7%)
Vancomycin	2 (2.1%)
Aminoglykosides	0
Amphotericin B	0
Other	4 (4.2%)

**Table 2:** Distribution of predefined variables in surviving and nonsurviving patients.

Variable	Survival	Death	*p* value
Gender (females in %)	47.1	43.6	0.79
Age (mean years+/−SD)	82.4+/−1.7	79+/−1.2	0.29
In-hospital stay (means days+/−SD)	14.9+/−2.8	22.4+/−2	0.051
AKIN stage (1/2/3 in %)	11.8/35.2/52.9	21.8/14/37.2	0.43
Initial serum creatinine (micro-Mol/L+/−SD)	143+/−281	196+/−273	0.74
Peak serum creatinine (micro-Mol/L+/−SD)	401.6+/−38.3	335.6+/−17.6	0.07 d
Serum creatinine before discharge (micro-Mol/L+/−SD)	355.7+/−45.2	189+/−12.4	**<0.001**
Initial serum sodium (mMol/L+/−SD)	138.3+/−1.0	137.4+/−0.5	0.81
Minimal serum sodium (mMol/L+/−SD)	136.1+/−1.2	134.9+/−0.7	0.57
Peak serum sodium (mMol/L+/−SD)	143.7+/−1.9	144.1+/−0.5	0.6
Initial serum potassium (mMol/L+/−SD)	5.2+/−0.2	4.8+/−0.1	0.2
Minimal serum potassium (mMol/L+/−SD)	4.2+/−0.2	3.6+/−0.07	**0.03**
Peak serum potassium (mMol/L+/−SD)	5.5+/−0.26	5.2+/−0.08	0.12
Initial NT-proBNP (pg/mL)	16,766+/−3,475	9,270+/−1,429	**0.015**
Peak NT-proBNP (pg/mL)	20,602+/−3,292	10,687+/−1,319	**0.005**
Initial CRP (mg/L+/−SD)	58+/−15.1	49.9+/−7.7	0.45
Peak CRP (mg/L+/−SD)	165.6+/−21	127.3+/−12.2	0.058
Vasopressors (%)	11.8	7.7	0.58
Invasive ventilation (%)	23.5	10.3	0.13
Arterial hypertension (%)	100	89.7	0.16
Diabetes (%)	76.5	52.6	0.07
Obesity (%)	43.8	57.7	0.3
Preexisting CHF (%)	29.4	26.9	0.83
Preexisting CAD (%)	35.3	42.3	0.59
COPD (%)	23.5	17.9	0.59
Hyperuricemia (%)	23.5	24.4	0.94
History of cancer (%)	41.2	24.4	0.15

**Table 3:** Distribution of predefined variables in patients with versus without the need for dialysis therapy.

Variable	No dialysis	Dialysis	*p* value
Gender (females in %)	46.8	33.3	0.3
Age (mean years+/−SD)	80.1+/−1.1	77.6+/−3	0.73
In-hospital stay (means days+/−SD)	19.5+/−1.9	27.6+/−4.2	**0.012**
AKIN stage (1/2/3 in %)	23.4/45.5/31.2	5.6/16.7/77.8	**0.001**
Initial serum creatinine (micro-Mol/L+/−SD)	196.6+/−12.8	378.2+/−58.8	**<0.001**
Peak serum creatinine (micro-Mol/L+/−SD)	317.6+/−12.9	474.6+/−56.5	**0.003**
Serum creatinine before discharge (micro-Mol/L+/−SD)	204.3+/−13.6	281.1+/−47.8	0.158
Initial serum sodium (mMol/L+/−SD)	137.3+/−0.7	138.8+/−1.5	0.49
Minimal serum sodium (mMol/L+/−SD)	134.9+/−0.7	136+/−0.8	0.58
Peak serum sodium (mMol/L+/−SD)	143.8+/−0.6	145.4+/−0.7	0.061
Initial serum potassium (mMol/L+/−SD)	4.8+/−0.1	5.3+/−0.2	**0.04**
Minimal serum potassium (mMol/L+/−SD)	3.7+/−0.08	3.7+/−0.09	0.72
Peak serum potassium (mMol/L+/−SD)	5.1+/−0.09	5.6+/−0.2	0.06
Initial NT-proBNP (pg/mL)	8,953+/−1,304	19,322+/−4,310	**0.02**
Peak NT-proBNP (pg/mL)	10,530+/−1,295	19,370+/−3,348	**0.018**
Initial CRP (mg/L+/−SD)	54.1+/−7.1	39.7+/−19.1	**0.034**
Peak CRP (mg/L+/−SD)	135.6+/−11.7	127.9+/−27.9	0.5
Vasopressors (%)	9.1	5.6	0.62
Invasive ventilation (%)	10.4	22.2	0.17
Arterial hypertension (%)	89.6	100	0.15
Diabetes (%)	55.8	61.1	0.68
Obesity (%)	51.3	72.2	0.1
Preexisting CHF (%)	27.3	27.8	0.96
Preexisting CAD (%)	35.1	66.7	**0.014**
COPD (%)	22.1	5.6	0.1
Hyperuricemia (%)	22.1	33.3	0.31
History of cancer (%)	29.9	16.7	0.25

**Table 4:** Distribution of all variables in subjects without versus with incomplete or complete recovery of kidney function.

Variable	No recovery	Incomplete recovery	Complete recovery	*p* value
Gender (females in %)	44.2	44.4	44.2	1
Age (mean years+/−SD)	79.9+/−1.3	84.3+/−2.3	78+/−2	0.35
In-hospital stay (means days+/−SD)	18.8+/−2.4	18.3+/−1.9	25.3+/−3.1	**0.037**
AKIN stage (1/2/3 in %)	23.1/48.1/28.8	33.3/22.2/44.4	11.8/32.4/55.9	0.09
Initial serum creatinine (micro-Mol/L+/−SD)	194.5+/−16.6	162.1+/−13.1	305+/−36.1	**0.011**
Peak serum creatinine (micro-Mol/L+/−SD)	337.2+/−19.2	313.7+/−30.9	372.2+/−33.1	0.75
Serum creatinine before discharge (micro-Mol/L+/−SD)	280.3+/−20.6	158.3+/−12.9	140.8+/−16.4	**<0.001**
Initial serum sodium (mMol/L+/−SD)	139.1+/−0.8	138.6+/−1.5	135.1+/−1.2	**0.029**
Minimal serum sodium (mMol/L+/−SD)	136.1+/−0.86	136+/−1.1	133.3+/−1	0.12
Peak serum sodium (mMol/L+/−SD)	143.4+/−0.8	144.1+/−0.92	145+/−0.87	0.22
Initial serum potassium (mMol/L+/−SD)	4.8+/−0.8	5.1+/−0.12	5+/−0.2	0.56
Minimal serum potassium (mMol/L+/−SD)	3.9+/−0.11	3.6+/−0.17	3.5+/−0.1	**0.031**
Peak serum potassium (mMol/L+/−SD)	5.2+/−0.1	5.2+/−0.15	5.38+/−0.14	0.61
Initial NT-proBNP (pg/mL)	10,319+/−1,770	13,615+/−3,697	9,501+/−2,616	0.41
Peak NT-proBNP (pg/mL)	12,822+/−1,859	17,263+/−3,967	10,181+/−1,955	0.18
Initial CRP (mg/L+/−SD)	40.5+/−6.4	78+/−23.1	61.2+/−15.2	0.43
Peak CRP (mg/L+/−SD)	124.2+/−13.5	154.6+/−27	143.9+/−20.9	0.45
Vasopressors (%)	9.6	0	8.8	0.62
Invasive ventilation (%)	17.3	0	8.8	0.4
Arterial hypertension (%)	92.3	100	88.2	0.51
Diabetes (%)	67.3	33.3	47.1	0.06
Obesity (%)	58.8	22.2	58.8	0.11
Preexisting CHF (%)	23.1	44.4	29.4	0.39
Preexisting CAD (%)	38.5	33.3	47.1	0.64
COPD (%)	21.1	33.3	11.8	0.24
Hyperuricemia (%)	21.2	22.2	29.4	0.67
History of cancer (%)	28.8	11.1	29.4	0.51

## Data Availability

The data supporting the findings of this study are available from the corresponding author upon reasonable request: daniel.patschan@mhb-fontane.de.
